# Supplementary material for: Enhanced Photocatalytic Activity and Stability of Bi2WO6 – TiO2-N Nanocomposites in the Oxidation of Volatile Pollutants
Source: Nanomaterials (Basel). 2022 Jan 23;12(3):359. doi: 10.3390/nano12030359 (PMC8838994; doi:10.3390/nano12030359)
Supplement: Supplementary file 1 [file nanomaterials-12-00359-s001.zip › nanomaterials-1517953-supplementary.pdf]

nanomaterials

Supplementary Materials for:

Enhanced Photocatalytic Activity and Stability of Bi_2WO_6 – TiO_2-N Nanocomposites in the Oxidation of Volatile Pollutants

Nikita Kovalevskiy [1,2], Svetlana Cherepanova [1], Evgeny Gerasimov [1], Mikhail Lyulyukin [1], Maria Solovyeva [1], Igor Prosvirin [1], Denis Kozlov [1] and Dmitry Selishchev [1,2,*]

[1] Boreskov Institute of Catalysis, 630090 Novosibirsk, Russia; nikita@catalysis.ru (N.K.), svch@catalysis.ru (S.C.)., gerasimov@catalysis.ru (E.G.), lyulyukin@catalysis.ru (M.L.), smi@catalysis.ru (M.S.), prosvirin@catalysis.ru (I.P.), kdv@catalysis.ru (D.K.), selishev@catalysis.ru (D.S.)
[2] Research and Educational Center "Institute of Chemical Technologies", Novosibirsk State University, 630090 Novosibirsk, Russia
* Correspondence: selishev@catalysis.ru; Tel.: +7-3833269429

Experimental setup for measurement of photocatalytic activity

The synthesized samples were tested in the oxidation of acetone vapor under UV and visible light in a continuous-flow set-up. The schematic diagram of the set-up is shown in Figure S1.

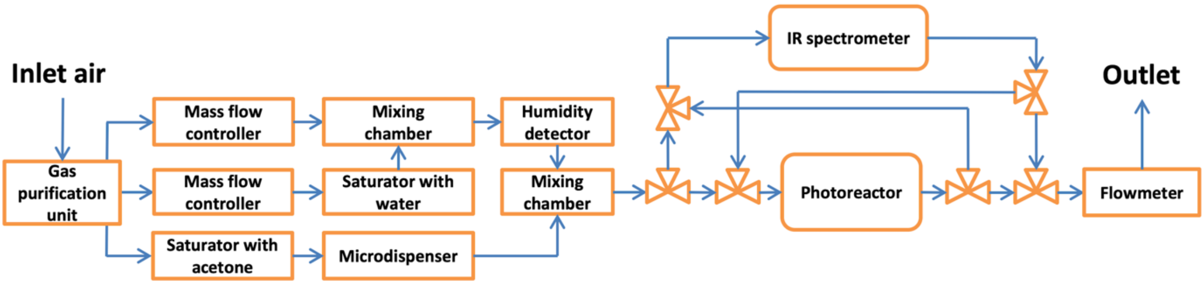

Figure S1. Schematic diagram of the set-up used for the photocatalytic experiments.

The set-up had a special valve system, which allows for analyzing the inlet and outlet reaction mixtures alternately using an FTIR spectrometer FT-801 from Simex LLC (Russia) equipped with an IR long-path gas cell (Infrared Analysis Inc., USA). During the analysis of inlet, the gas from a mixing chamber flows firstly through the IR cell and then goes to the photoreactor. The photocatalyst was deposited on a round glass plate in the area of 9.1 cm^2 and placed into the photoreactor. The surface density of the photocatalyst on the glass was 20 mg cm^{-2}. In the case of outlet analysis, the gas flows through the photoreactor and then through the IR cell. The time interval for the analysis of both the inlet and outlet during the experiments was 10 min. The other experimental parameters were as follows: the reactor temperature is 40 ± 0.1 °C, the relative humidity is 19 ± 1%, the volume flow rate is 0.067 ± 0.001 L min^{-1}.

Acetone was selected as a test organic substrate for the photocatalytic experiments due to it does not cause the deactivation of photocatalyst and is completely oxidized to CO_2 and water without gaseous intermediates that allows for valid evaluation of photocatalytic activity and light utilization efficiency using the rate of CO_2 formation due to CO_2 has much higher attenuation coefficient than acetone and results in more accurate values. To monitor the concentrations of acetone and CO_2 during the experiment, the IR spectra were collected periodically every 30 s. The quantitative analysis was performed by the integration of collected IR spectra using the Beer-Lambert law as follows:

$$\int_{\omega_1}^{\omega_2} A(\omega)\,d\omega = \varepsilon \times l \times C \quad (S1)$$

where $A(\omega) = \lg\left(\frac{I_0(\omega)}{I(\omega)}\right)$ is the absorbance, ω_1 and ω_2 are the limits of the corresponding absorption bands (cm^{-1}), ε is the attenuation coefficient (μmol^{-1} L cm^{-2}), l is the optical path length (cm), and C is the concentration of a substance in the gas phase (μmol L^{-1}). The regions for calculation were selected as follows: 1160–1265 cm^{-1} for acetone and 2200–2400 cm^{-1} for CO_2. The attenuation coefficients for each substance were calculated from the calibration data.

Before the evaluation of photocatalytic activity, the adsorption-desorption equilibrium of acetone on the photocatalyst was achieved until no difference in inlet and outlet acetone concentrations was observed. After that, a light source was turned on and the photocatalytic activity was evaluated. A high-power UV LED with a maximum at 371 nm and blue LED with a maximum at 450 nm were used for the photocatalyst irradiation. Figure S2 shows the emission spectra of the diodes that are measured using an ILT 950 spectroradiometer from International Light Technologies Inc. (USA). The total irradiance was 9.7 mW/cm^2 for UV-LED and 164 mW/cm^2 for Blue-LED. The total irradiance was 10 mW cm^{-2} for UV LED and 160 mW cm^{-2} for blue LED.

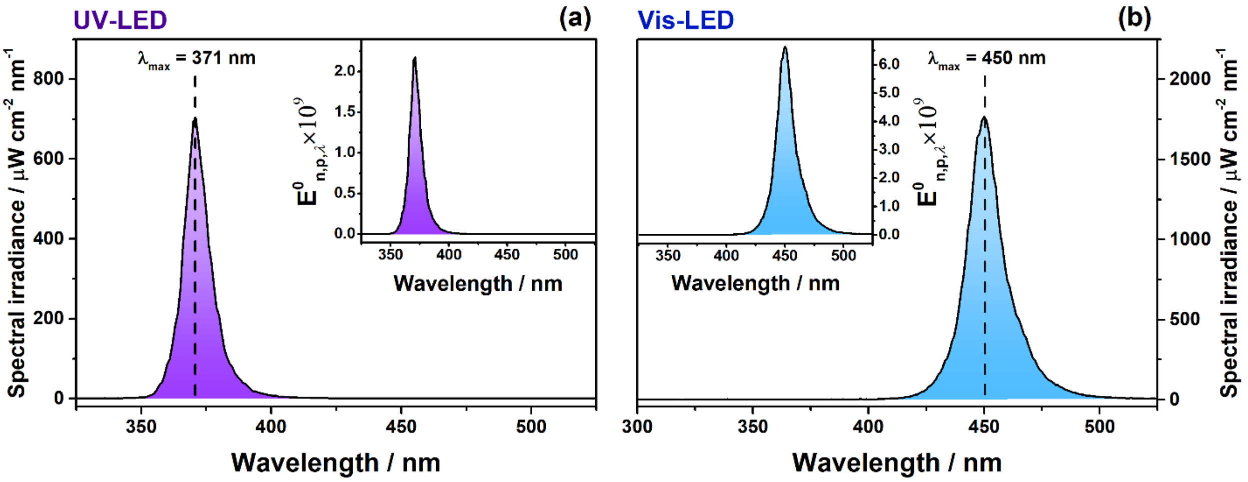

Figure S2. Emission spectra of the diodes used for irradiation of photocatalysts.

The photocatalytic activity of the catalyst synthesized was evaluated as the rate of CO_2 formation during the acetone oxidation. The CO_2 rate can be expressed as follows:

$$W_{CO_2} = \Delta C_{CO_2} \times U$$

where W_{CO_2} is the steady-state rate of CO_2 formation (μmol min^{-1}), ΔC_{CO_2} is the difference in the outlet and inlet CO_2 concentration (μmol L^{-1}), U is the volume flow rate (L min^{-1}). Based on the statistics of many experiments, a total error in measuring the CO_2 rate using the set-up does not exceed 10%.

Analysis of XRD pattern for synthesized Bi_2WO_6

The experimental X-ray diffraction pattern generally corresponds to the orthorhombic Bi_2WO_6 (PDF#39-0256, space group: Pbca, a = 5.4568 Å, b = 16.4355 Å, c = 5.4382 Å), but a peak at 2θ~33° contains a shoulder on the small-angle slope of the peak (shown by an arrow in Figure S3(A)). It should be also noted that peaks at 2θ~47° and 2θ~56° have a

wide base and a narrow top ("triangular" peak shape), which does not allow them to be well fitted by the pseudoVoight function. This peak shape is usually the result of a bimodal particle size distribution. In addition, such shape of the peaks is possible if the peaks contain several diffraction lines, where the central diffraction line is the most intense. In our case, peaks with a narrow top and a wide base just consist of several lines. However, the diffraction lines are located very close to each other because lattice constants a, c and reduced parameter b/3 have very similar values (a~b/3~c). Such a close arrangement of diffraction lines does not lead to a "triangular" shape of peaks in the case of an isotropic shape of particles. At the same time, such a shape of the peaks may indicate a significant anisotropy of the particle sizes.

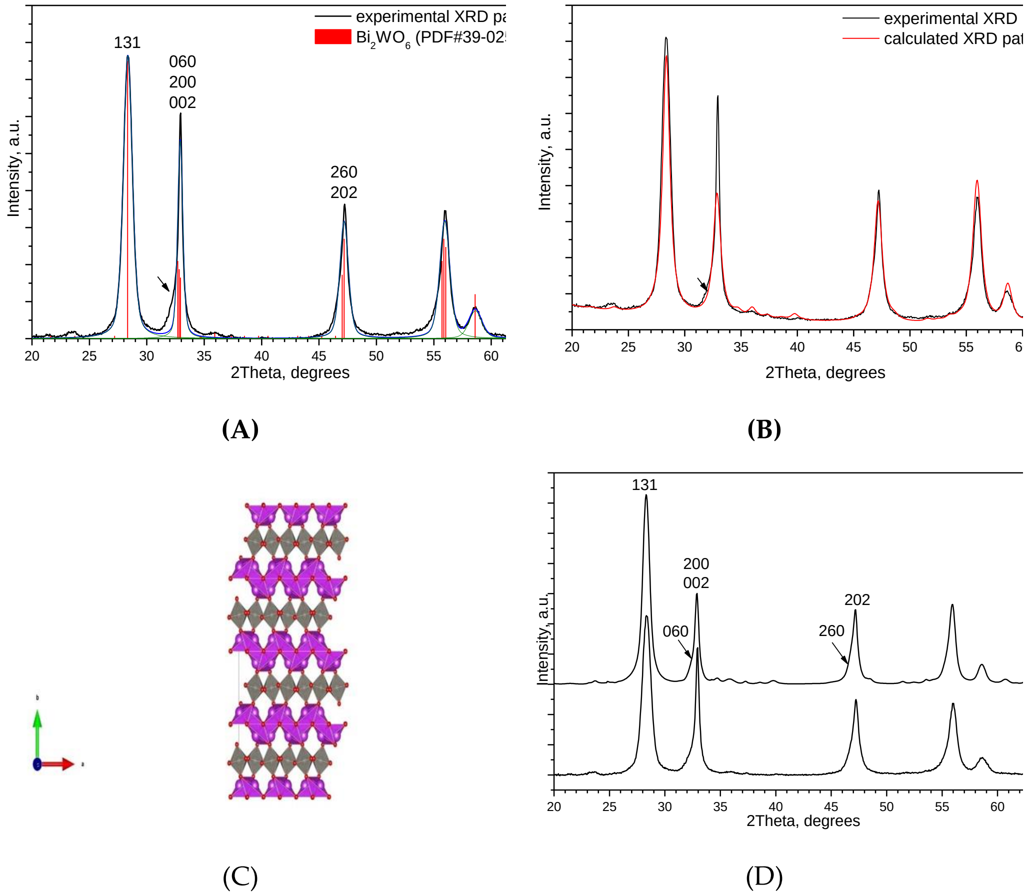

Figure S3. Analysis of XRD patterns: (**A**) Experimental XRD pattern of Bi_2WO_6: decomposition, peak approximation by pseudoVoight functions; (**B**) Rietveld refinement (spherical particles); (**C**) Structure of Bi_2WO_6; (**D**) a) XRD pattern calculated on the basis of model of Bi_2WO_6 particles of disk-like shape (D = 30 nm, H = 6.6 nm); b) experimental XRD pattern of Bi_2WO_6.

The Rietveld analysis of the diffraction data was carried out using the TOPAS 4-2 program (Bruker, Germany). Generally, the calculated X-ray diffraction pattern fits the experimental one (Figure S3(B)). However, the peak in the range 2θ~31–35° in the calculated X-ray diffraction pattern is symmetric, while in the experimental one it is asymmetric. Most likely, this is due to the limitation of the model by isotropic particle sizes (spherical particles). An average particle size was about of ~ 11 nm.

The structure of Bi_2WO_6 is layered, the layers are located perpendicular to the **b** direction (Figure S3(C)). Therefore, it can be assumed that the particles have a plate-like shape, i.e. the average dimensions in the direction perpendicular to the layers (**b**) are significantly smaller than in the plane of the layers (**a** and **c**). And, X-ray diffraction patterns for anisotropic Bi_2WO_6 particles were calculated using the program [58,59]. The program allows calculating X-ray diffraction patterns based on models of partially disordered

nanocrystals with anisotropic sizes. The varied parameters were diameter D and height H of disk-like particles. The best correspondence with the experimental diffraction pattern was obtained for disk-like particles with D ~ 30 nm, H = 6.6nm (Figure S3(D)). In this case, X-ray diffraction pattern is better fitted on the basis of the plate-like particle model as compared to the spherical one (Figure S3(B)). In addition, the lamellar shape of the particles is confirmed by microscopy.

Stability of N-doped TiO_2 photocatalyst

The photocatalytic activity of pristine TiO_2-N is gradually decreased under long-term irradiation, but after several days of irradiation its activity reaches a permanent level, namely, ca. 40% of initial value, and does not change over further time.

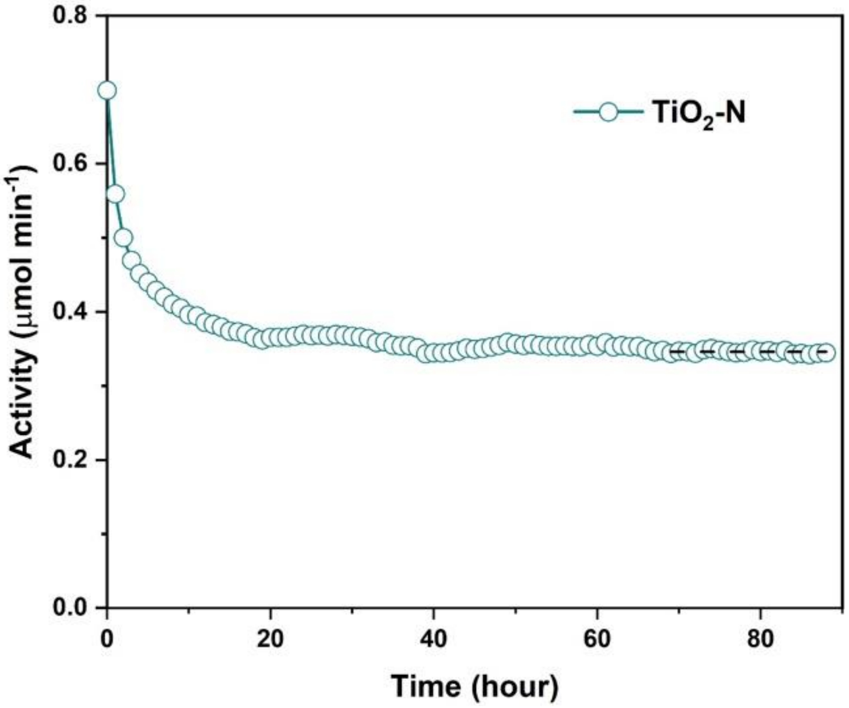

Figure S4. Long-term stability of TiO_2-N under blue light of 160 mW cm^{-2}.
